# Association between night-shift work and sleep quality among nurses

**DOI:** 10.1192/j.eurpsy.2025.2338

**Published:** 2025-08-26

**Authors:** N. Rmadi, A. Hrairi, F. Bouzid, F. Dhouib, A. Kchaou, M. Hajjaji, K. Jmal Hammami

**Affiliations:** 1 Occupational medicine department, Hedi chaker university hospital, University of Sfax; 2 Family medicine department, University of Sfax, Sfax, Tunisia

## Abstract

**Introduction:**

Night-shift work significantly impacts sleep quality among nurses, leading to various adverse health outcomes.

**Objectives:**

This study aimed to assess the link between night-shift work and sleep quality among nurses.

**Methods:**

The study was conducted with a sample of nurses in university hospitals of Sfax. Two groups of staff were defined based on their work schedule: the first group(G1) consisted of those working day shifts, either a regular morning schedule or alternating between morning and afternoon shifts, while the second group (G2) included those working night shifts, either fixed night shifts or alternating between morning, afternoon, and night shifts. Data collection was carried out using an anonymous self-questionnaire developed via an online interface hosted on Google Forms. Sleep disorders were screened using the validated Arabic version of the Pittsburgh Sleep Quality Index (PSQI).

**Results:**

The study population consisted of 114 nurses, with 37 nurses in G1 and 77 in G2. The average age of the workers was 33.8 years ± 7 years with extremes of 23 and 55 years. The average duration of night work was 5.9 years ± 4.64 years, ranging from a minimum of 1 year to a maximum of 25 years. The overall PSQI scale score was on average 6.86 ± 3.2. Based on this scale, 62% were classified as poor sleepers. In bivariate analysis, night-shift work was associated with a bad sleeper profile (p= 0.027, OR=2.44, IC95% [1.09-5;46]). However, day-shift work protected from the bad sleeper profile (p=0.04, OR=0.4, IC95% [0.18-0.91]).

**Conclusions:**

The study highlights the negative association between night-shift work and sleep quality among nurses. it is essential for healthcare organizations to implement strategies that address the unique challenges faced by night-shift nurses, such as promoting better sleep hygiene and providing support resources.

**Disclosure of Interest:**

None Declared

